# Flow-Through Catalytic Reactors Based on Metal Nanoparticles Immobilized within Porous Polymeric Gels and Surfaces/Hollows of Polymeric Membranes

**DOI:** 10.3390/polym12030572

**Published:** 2020-03-04

**Authors:** Sarkyt E. Kudaibergenov, Gulzhian I. Dzhardimalieva

**Affiliations:** 1 Satbayev University, Laboratory of Engineering Profile, Almaty 050013, Kazakhstan; 2 Institute of Polymer Materials and Technology, Almaty 050019, Kazakhstan; 3 Institute of Problems of Chemical Physics Russian Academy of Sciences, Chernogolovka, Moscow 142432, Russia; dzhardim@icp.ac.ru

**Keywords:** flow-through catalytic reactor, nano-, micro- and macrosized porous polymeric gels, polymeric membranes, metal nanoparticles, catalytic reactions

## Abstract

State-of-the-art of flow-through catalytic reactors based on metal nanoparticles immobilized within the pores of nano-, micro- and macrosized polymeric gels and in the surface or hollow of polymeric membranes is discussed in this mini-review. The unique advantages of continuous flow-through nanocatalysis over the traditional batch-type analog are high activity, selectivity, productivity, recyclability, continuous operation, and purity of reaction products etc. The methods of fabrication of polymeric carriers and immobilization technique for metal nanoparticles on the surface of porous or hollow structures are considered. Several catalytic model reactions comprising of hydrolysis, decomposition, hydrogenation, oxidation, Suzuki coupling and enzymatic reactions in the flow system are exemplified. Realization of “on-off” switching mechanism for regulation of the rate of catalytic process through controlling the mass transfers of reactants in liquid media with the help of stimuli-responsive polymers is demonstrated. Comparative analysis of the efficiency of different flow-through catalytic reactors for various reactions is also surveyed.

## 1. Introduction

Catalysis by functional polymers themselves [1], polymer-metal complexes [2], polymer-immobilized clasters and metal nanoparticles [3,4] as well as polymer-protected and gel-immobilized metal nanoparticles [5,6,7] is intensively developed subject at the interface between such disciplines as macromolecular chemistry, catalysis, and nanotechnology [8,9]. However the polymer-based flow-through catalytic systems were intensively developed only in the past decade. The flow-through catalytic reactor is defined as a catalytic system enabling continuously passing the reactants into one end and obtaining the products from the other end [10,11]. There are several types of flow-through catalytic reactors derived from nano-, micro- and macroporous inorganic and polymeric materials, in particular hydrogels and cryogels [12], thin membranes and layer-by-layer (LbL) films [13], hollow fibers [14] or tubular construction [15]. An artificial catalytic flow reactor constructed from the porous inorganic and polymeric materials containing metal nanoparticles can mimic the function of a living system where the chemical synthesis proceeds through capillaries and cells [16]. In our mind, the concept of “green chemistry” in the context of catalytic chemistry means reaction behavior in mild conditions, e.g. preferentially in aqueous solution, at atmospheric pressure and room temperature including the easy separation of products from catalysts. These requirements can successfully be realized in case of flow-through catalytic reactors made of porous polymeric materials within which metal nanoparticles or enzymes are immobilized. From practical point of view the flow-through catalytic reactors based on porous gel- and membrane-immobilized metal nanoparticles provide a new platform for production of fine chemicals, industrial products and treatment of wastewater from organic pollutants.

## 2. Flow-Through Catalytic Reactors Fabricated from Porous Hydrogels and Metal Nanoparticles

**Figure 1:**
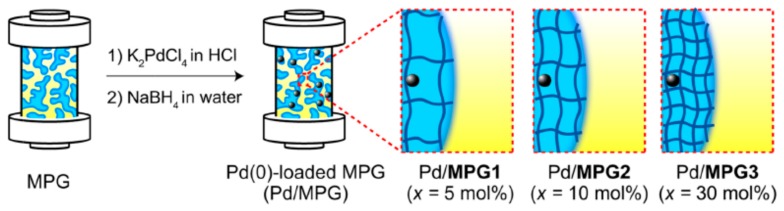
Immobilization of PdNPs into the gel pores of MPGs. (Reprinted from [16]).

Monolithic porous hydrogels (MPGs) with interconnected capillaries were prepared by copolymerization of N-isopropylacrylamide (NIPAm) and N-(3-dimethylaminopropyl)methacrylamide (DMAPM) in the presence of N,N-methylenebisacrylamide (MBAAm) as a crosslinker and loaded with Pd(0) nanoparticles (PdNPs) [16]. Three types of PdNPs/MPGs abbreviated as PdNPs/MPG-1, PdNPs/MPG-2 and PdNPs/MPG-3 at different crosslinking degrees 5, 10 and 30 mol.% MBAA were tested as flow reactors for Suzuki coupling reaction as a model system (Figure 1). 

The advantages of PdNPs/MPGs were compared with PdNPs-immobilized porous glass membrane, silica and carbon particles. The influence of the gel structure on molecular transport of substrates was also studied. The pore sizes of PdNPs/MPGs varied from several hundred nanometers to several micrometers while the average size of immobilized PdNPs was equal to 2.0–2.4 nm for all PdNPs/MPGs samples. The temperature dependent volume-phase transition of MPGs caused by NIPAAm was suppressed by increasing the MBAA content. The turnover numbers (TONs) of PdNPs-loaded supports at constant flow rate (at *t* = 0.5 h) and turnover frequencies (TOFs) (at *t* = 0.1 h) were estimated along with permeation of the substrate during 30 days together with Pd leaching (Table 1).

**Figure 2:**
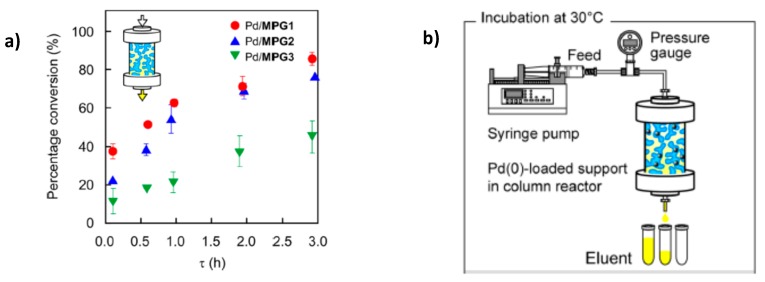
Conversion of Suzuki coupling reaction (**a**) in the flow reactor PdNPs/MPGs (**b**). (Reprinted from [16]).

The kinetic studies of Suzuki reaction are shown in Figure 2. It is seen that the conversion of Suzuki coupling reaction increases in the order PdNPs/MPG-1 > PdNPs/MPG-2 > PdNPs/MPG-3. In spite of the fact that the surface areas of capillaries in PdNPs/MPGs increase with increasing MBAAm content, the molecular transport of reagents is not retarded by surface capillaries. Thus PdNPs/MPGs flow reactor is a new strategy for chemical synthesis and has a significant impact to expand our fundamental understanding of catalytic reactions. 

**Figure 3:**
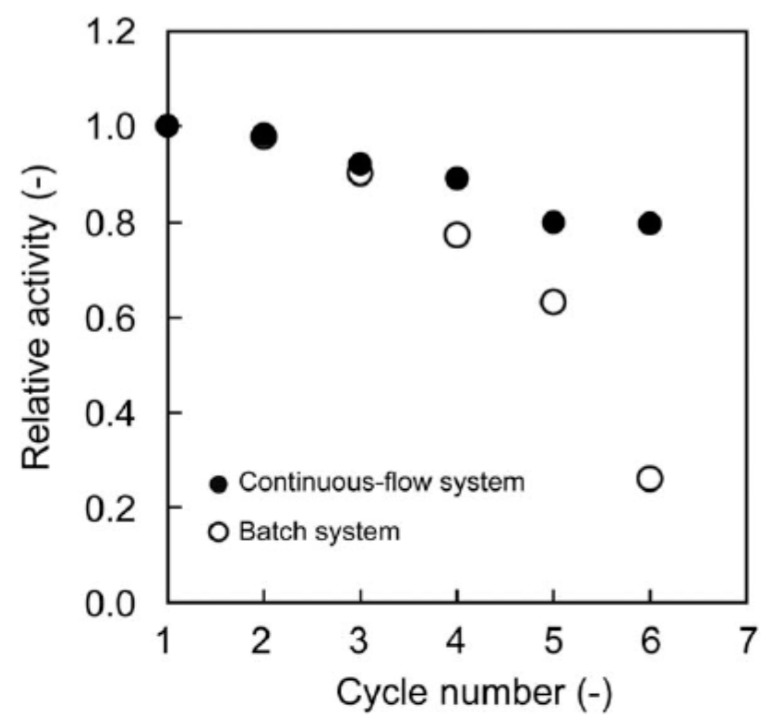
Storage stability of PdNPs-loaded membrane in continuous-flow (●) and batch (o) system for Suzuki coupling reaction. (Reprinted from Ref.17).

Continuous-flow Suzuki coupling reaction was carried out with the help of PdNPs-loaded terpolymeric nanogel particles composed of NIPAm, N-3-dimethylaminopropylmethacrylamide (DMAPM) and N-(3-aminopropyl)methacrylamide hydrochloride (APM) crosslinked by MBAAm [17]. Nanogel particles containing PdNPs were deposited to a filter paper consisting of activated by amine groups SiO_2_ and used as a combination of 4 sheets membrane reactor for the Suzuki coupling reaction between phenylboronic acid and 4-iodobenzoic acid at 60 °C. The feed solution was passed through the membrane reactor at a flow rate of 1.0 mL·h^−1^ (t = 2.2 h) until the conversion degree of final product – 4-phenylbenzoic acid reached a steady state. The rate constants *k_obs_* in the batch and continuous-flow systems were equal to 1.6 and 0.46 h^−1^ respectively. As it is seen from Figure 3 the relative activity of the membrane-loaded PdNPs catalyst in the continuios-flow system is much higher than in the batch system. Moreover the long-term stability of PdNPs-loaded membrane reactor TON reached up to 1200 during 6 days without considerable leaching of PdNPs (less than 1%) and maintaining the fibrous structure of the used membrane. However at initial time period the conversion time into 4-phenylbenzoic acid in batch system is proved higher than continuous-flow membrane catalyst. This is attributed to lower density of PdNPs in nanogel-based membrane reactor. But after 8–10 h, the conversion percentage of 4-phenylbenzoic acid becomes flattened for both system and is approximated to 100%. 

**Figure 4:**
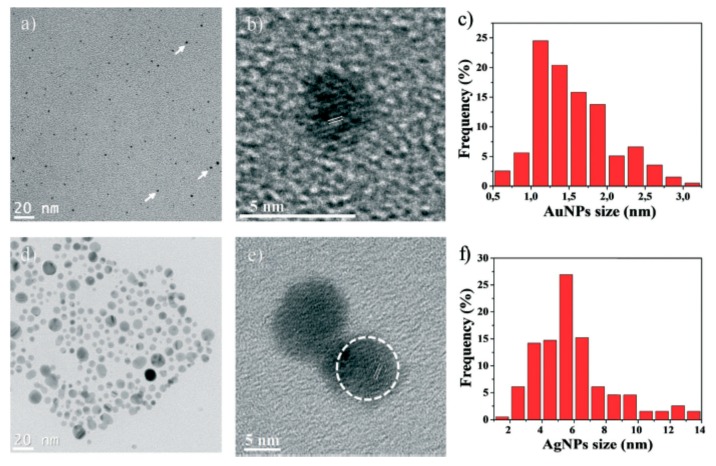
Nanoparticles characterization: TEM micrographs of AuNPs (**a**), AgNPs (**d**); HRTEM images of single AuNPs (**b**) and AgNPs (**e**) with corresponding lattice spacing; Size distribution histogram of AuNPs (**c**) and AgNPs (**f**) (calculated by measuring ≈ 200 AuNPs and AgNPs). (Reprinted from [19]–Reproduced by permission of The Royal Society of Chemistry).

Remarkable experiments were carried out by Gancheva and Virgilio [18,19] on the synthesis and characterization of thermo-responsive macroporous PNIPAm hydrogel monoliths with precisely controlled pore size, surface area, pore volume, within which PdNPs, gold (AuNPs) and silver (AgNPs) nanoparticles were embedded to catalyze the reduction of 4-nitrophenol (4-NP) to 4-aminophenol (4-AP) in flow-through reactor conditions. Distribution, size and morphology of AgNPs and AuNPs in the pores of PNIPAm show that the nanoparticles are mostly spherical and well-dispersed with an average diameter of 1.6 ± 0.5 nm (AuNPs) and 5.9 ± 2.4 nm (AgNPs) (Figure 4).

The macroporous hydrogel-metal nanoparticles were tested for hydrogenation of 4-NP as a flow-through catalytic reactors passing the 4-NP solution through the monolith at a flow rate of 20, 80, 120, 200 and 250 mL·h^−1^. Depending on the flow rate the conversion of 4-NP varied from 7 to 98%. The following advantages of macroporous monolith hydrogels as continuous-mode microreactors are outlined: (1) Easy preparation technique, (2) Regulation of microstructure and porosity; (3) Excellent permeability with respect to reactants and products; (4) High stability of metal nanoparticles. The main disadvantage is a weak mechanical characteristic of macroporous monolith hydrogels that may be overcome by forming interpenetrating gel networks or by modifying them with porous inorganic materials.

**Figure 5:**
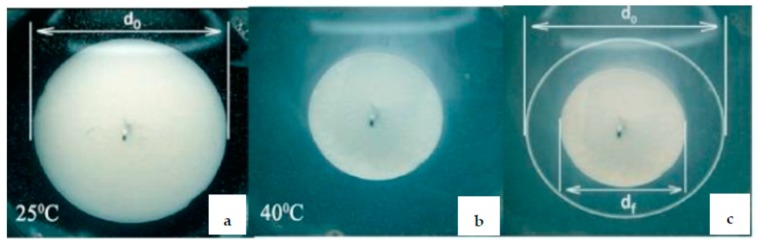
The diameter of PNIPAm porous hydrogel loaded by PVP-PdNPs at 25 °C (**a**) and 40 °C (**b**) demonstrating the swelling-shrinking of PNIPAm hydrogel (**c**). (Reprinted from [21]).

The use of thermo-responsive hydrogels in whole and poly(N-isopropylacrylamide) (PNIPAm) in particular, in catalytic processes is well known [20,21]. Poly-N-vinylpyrrolidone-protected palladium nanoparticles (PVP-PdNPs) immobilized within the thermo-responsive PNIPAm hydrogel demonstrated “on-off” mechanism in the course of 2-propen-1-ol hydrogenation to propanol [21,22]. Due to porous structure and swelling-deswelling behavior of PNIPAM hydrogel at temperature interval 25–45 °C the outflow or inflow of PVP-PdNPs from or to hydrogel matrix takes place (Figure 5). 

**Figure 6:**
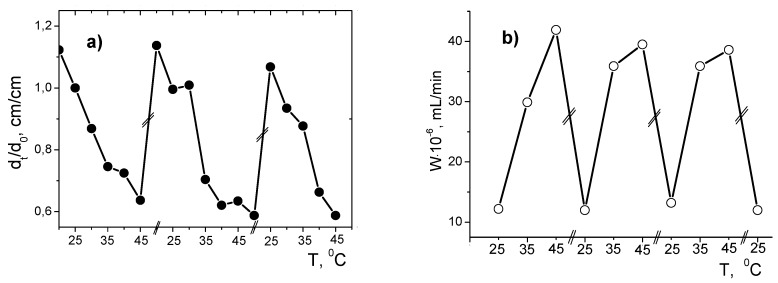
Temperature dependent cyclic changes in size (**a**) and the rate of hydrogenation of 2-propen-1-ol by PNIPAm/PVP-PdNPs hydrogel (**b**). (Reprinted from [21]).

Periodic opening and closing of PNIPAM hydrogel pores act as a “nanogate” in flow-through catalytic reactor and leads to periodic increase or decrease of the pore size (d_t_/d_0_) and hydrogenation rate (W) of 2-propen-1-ol (Figure 6).

## 3. Flow-Through Catalytic Reactors Based on Macroporous Cryogels and Metal Nanoparticles

**Figure 7:**
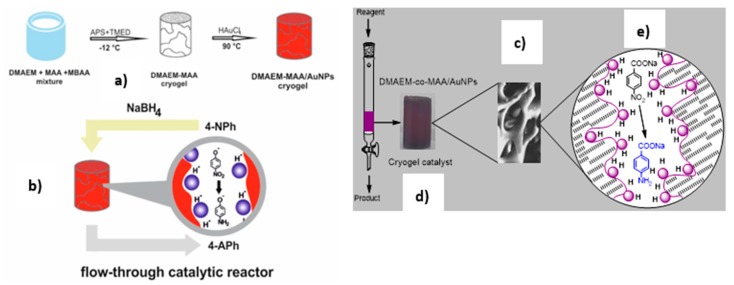
Fabrication of macroporous amphoteric cryogel based on N,N-dimethylaminoethylmetacrylate and methacrylic acid (DMAEM-co-MAA) with immobilized AuNPs (**a**) and schematic representation of monolith flow-through catalytic reactor used for hydrogenation of 4-NP (**b**) and p-NBA (**c**) over DMAEM-co-MAA/AuNPs catalysts. The violet colored sample corresponds to macroporous cryogel DMAEM-co-MAA containing AuNPs (**d**) while the violet dots are schematic image of AuNPs (**e**) in cryogel pores (Reprinted from [7,26]).

As distinct from hydrogels, cryogels synthesized in cryogenic conditions can consist of interconnected macroporous matrices with the pore size from ~ 0.1 to 10 μm and supermacroporous matrices with the pore size in the range from several tens to several hundreds of micrometers [23,24,25]. Due to high surface/volume ratio and easy convection of liquids inside cryogel matrices, both macroporous and supermacroporous cryogels are proved to be the most favorable materials to design flow-through catalytic reactors [7,26,27] (Figure 7). 

Macroporous cryogels containing metal nanoparticles in pores can be used for catalytic reactions as batch-type and continuous-flow-type reactors. In the first case, a certain amount of cryogel catalyst is mixed with the substrate and reducing agent under stirring. In the latter case, the monolithic macroporous cryogel sample is replaced inside a glass tube and the reaction mixture is fed as forced feed or flows under gravity. Significant advantages of macroporous flow-through catalytic reactors over the batch-type mode are the simplicity, process automation, convenient monitoring of liquid stream (or speed control), easy regulation of the feed concentration and reaction temperature, fast analysis of the product, permanent loading of metal nanoparticles within cryogel pores, stability, high productivity, green reaction profile and safety [7,10,18,19,20,21,22,26].

The idea of the use of macroporous and supermacroporous cryogels as flow-through catalytic reactors was for the first time suggested in [12] and experimentally realized in [7,26,27,28,29,30] for hydrogenation of nitroaromatic compounds (Figure 7). Later on Sahiner et al. [31,32] using the same principles and glass column reactor repeated reduction of 4-nitrophenol (4-NP) to corresponding 4-aminophenol (4-AP) with the help of superporous cryogels containing various metal nanoparticles and were able to lower the activation energy (E_a_) of catalytic reduction of 4-NP considerably in comparison with similar studies reported in the literature.

Equimolar polyampholyte cryogel P(DMAEM-*co*-MAA) synthesized from acidic and basic monomers, such as N,N-dimethylaminoethylmethacrylate (DMAEM) and methacrylic acid (MAA) effectively reduces rhodium, palladium, gold and silver ions under heating conditions and forms fine well-dispersed metal nanoparticles without the use of any other reducing agents [30]. Moreover micron-sized cryogel matrix provides fast swelling during 0.5–2 min and high water flux [26]. Nonionic [33,34], anionic [35,36], and cationic [37] cryogel-immobilized metal nanoparticles were successfully used for decomposition of NaBH_4_ and hydrogenation of nitrogroup containing substrates in batch conditions.

Porous P(DMAEM-*co*-MAA) cryogel with immobilized AuNPs was used as a flow-through catalytic reactor in reduction of 4-NP and oxidation of D,L-dithiotreitol (DTT) [38,39,40]. The final hydrogenation product of 4-NP is 4-AP, while the final oxidation product of DTT is disulfide (DS). The kinetic parameters, turnover number (TON), turnover frequency (TOF) and activation energy of hydrogenation of 4-NP and oxidation of DTT were determined in these experiments (Table 2).

The catalytic reduction of *p*-nitrobenzoic acid (*p*-NBA) was performed by palladium (PdNPs) and gold nanoparticles (AuNPs) immobilized within P(DMAEM-*co*-MAA) cryogel matrix [41] (Figure 7). It should be noted that in the absence of immobilized metal nanoparticles the mixture of *p*-NBA and NaBH_4_ fluxed through the P(DMAEM-*co*-MAA) cryogel does not produce *p*-aminobenzoic acid (*p*-ABA) [42].

Hydrogenation of *p*-NBA yields at least 3 main products: 1) *p*-ABA, 2) *p,p’*-azodibenzoate and 3) sodium 4-(4-aminobenzamido)benzoate. In case of P(DMAEM-*co*-MAA)/PdNPs the formation of only *p*-ABA with conversion degree 40% at [*p*-NBA]:[NaBH_4_] = 1:50 mol/mol and 100% at [*p*-NBA]:[NaBH_4_] = 1:200 mol/mol is observed. According to the activation energy, TONs and TOFs values established in these experiments the efficiency of macroporous flow-through reactor (Table 2) is greater compared to the macroporous batch-type reactor (Table 3).

Last years polymeric macroporous cryogel- or polymeric membrane-based enzymatic reactors (or bioreactors) have received much attention due to the following advantages: (1) the simultaneous use of two or more enzymes, (2) the possibility to carry out reactions in immiscible solvents (biphasic system), (3) the conversion of high molecular weight substrates, (4) the operation in the condition of convection and not diffusion, and (5) the simultaneous formation, separation and concentration of the product [45]. The activity of several enzymes immobilized within microporous of cryogels as flow-through catalytic reactor is presented below.

Amyloglucosidase immobilized within poly(methyl methacrylate-glycidyl methacrylate) P(MMA-*co*-GMA) cryogels was used as a flow-through catalytic reactor for continuous glucose syrup production from starch [46]. Enzyme was attached to P(MMA-*co*-GMA) cryogels through covalent bonding with participation of epoxy groups of GMA and primary amine groups of amyloglucosidase.

To perform enzymatic reaction 1 wt.% starch solution was passed through the amyloglucosidase-immobilized cryogel column using a peristaltic pump with the flow rate of 1 mL·min^−1^. The condensation reaction between epoxy groups of P(MMA-*co*-GMA) cryogels and primary amino groups of amyloglucosidase leads to formation of covalent linkages. Immobilized amount of amyloglucosidase within cryogel matrix was equal to 146 mg·g^−1^ and showed 68% activity after 20th reuse. Optimal pH activity for both free and immobilized enzyme was found to be 5.0. Optimal temperature activity for free enzyme corresponds to 55 °C and it shifts to 65 °C in case of immobilized enzyme. The maximum reaction velocity *V_max_* and Michaelis-Menten constant K*_m_* of free and bound amyloglucosidase are summarized in Table 4. 

The decreased values of K*_m_* and *V_max_* of immobilized enzyme in comparison with free precursor may be accounted for restricted accessibility of some active sites of enzyme to bulky substrate. Due to interconnected and large porous structure and low pressure drop, such cryogel matrices can be used for continuous syrup production in industrial scale as demonstrated by Milosavic and coworkers [47]. A packed bed reactor containing immobilized enzyme produced continuously 1300 kg of glucose per 1 L of reactor volume during 4 weeks. 

Peroxidase immobilized poly(acrylamide) cryogels were prepared and used for removal of phenol, bisphenol A, guaiacol, pyrogallol, and catechol from aqueous solution [48]. Maximum peroxidase loading onto poly(acrylamide) cryogel was found to be 127.3 mg·g^−1^. Kinetic parameters of free and immobilized peroxidases were investigated along with the stability tests. Removal capacities of phenolic compounds were equal to 96.3% (phenol), 75.8% (bisphenol A), 79.7% (guaiacol), 64.9% (pyrogallol) and 71.0% (catechol). Thus, one can conclude that cryogel immobilized enzymes are effective system for industrial production of glucose and purification of wastewaters from the phenolic contaminants. 

## 4. Flow-Through Catalytic Reactors Designed by Modification of the Surface and Hollow of Polymeric Membranes with Metal Nanoparticles 

Comprehensive information on metal nanoparticles or nanosized metal oxides embedded in a matrix of polymeric membranes has been reviewed in [49]. In particular, various types of polymeric membranes impregnated with metal nanoparticles or metal oxides (Ag, Al, Fe, Mg, Si, Ti, Zr) that impact on the mechanical strength, thermal stability, permeability, selectivity, conductivity, and antiviral and antibacterial activity of membrane materials are outlined. Different categories of the membranes with their respective description are given. Application aspects of surveyed polymeric membranes incorporated with metal or metal oxides nanoparticles cover mostly liquid or gas separation, fuel cells, production of drinking water etc. This remarkable review may serve as prerequisite for fabrication of various types of effective catalysts based on metal nanoparticles-immobilized polymeric membranes and open a perspective insight into new generation of polymeric membrane catalytic system. 

**Figure 8:**
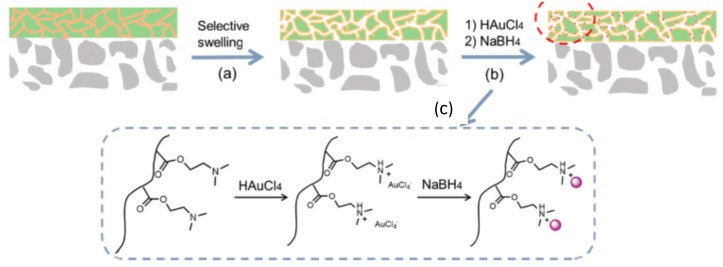
Selective swelling of PDMAEM-*b*-PS membrane (**a**), immobilization of gold nanoparticles on the surface of DMAEM-*b*-PS membrane (**b**), protonation of PDMAEM and reduction of [AuCl_4_]^−^ counterions to AuNPs (**c**). (Reproduced by permission of the Royal Society of Chemistry from [50]–Reproduced by permission of The Royal Society of Chemistry).

Zhou et al. [50] fabricated nanoporous membranes supporting the block copolymers of poly(2-dimethylaminoethyl methacrylate) (PDMAEM) and polystyrene (PS) (PDMAEM-*b*-PS) with immobilized gold nanoparticles (AuNPs) on the surface of macroporous poly(vinylidene fluoride) (PVDF) membranes (Figure 8). 

**Figure 9:**
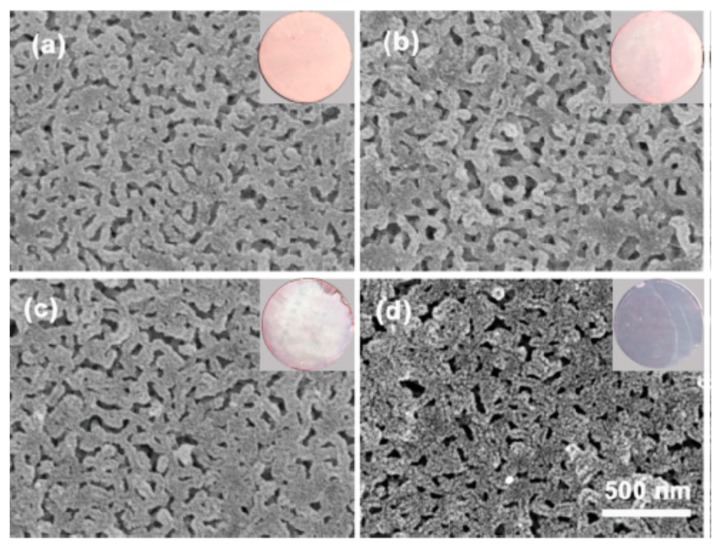
SEM and digital images of PDMAEM-*b*-PS/AuNPs membranes prepared with concentrations of HAuCl_4_ (**a**) 0.5 mg·mL^−1^; (**b**) 1.25 mg·mL^−1^; (**c**) 2.5 mg·mL^−1^; (**d**) 5.0 mg·mL^−1^. ((Reproduced by permission of the Royal Society of Chemistry from Ref. [50]–Reproduced by permission of The Royal Society of Chemistry).

Selective swelling of PDMAEM in hot ethanol followed by rapid evaporation of the solvent generated the interconnected nanopores with tunable pore size and geometry. Soaking of PDMAEM in HAuCl_4_ solution protonates tertiary amino groups replaced on the surface and pore walls of membrane while the [AuCl_4_]^−^ become as counterions. Further reduction of PDMAEM-b-PS/[AuCl_4_]^−^ by sodium boronhydride produces AuNPs-immobilized PDMAEM-*b*-PS/AuNPs membrane. Depending on HAuCl_4_ concentration the color of the membranes is varied from pink to dark purple due to the surface plasmon resonance of AuNPs (Figure 9). 

The flow-through catalytic reactor made of PDMAEM-*b*-PS/AuNPs membranes was tested for hydrogenation of 4-nitrophenol (4-NP) and degradation of Rhodamine B and methyl orange (MO). Conversion degree of 4-NP to 4-aminophenol (4-AP) reached up to 100% exhibiting excellent recyclability. The catalytic degradation of Rhodamine B and MO reached up to 91% and 88% respectively.

**Figure 10:**
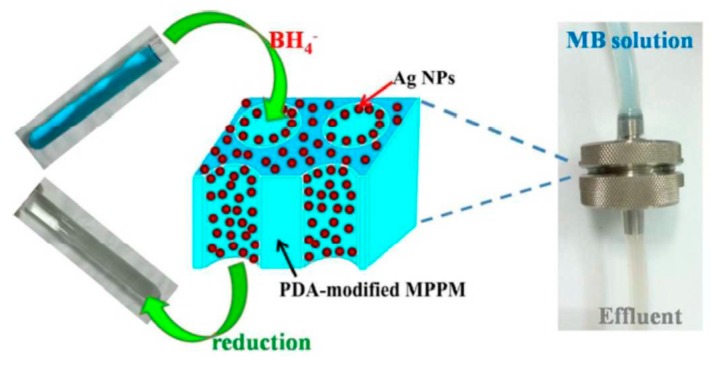
Reduction of MB with the AgNPs-immobilized MPPM in a flow-through membrane reactor. ((Reproduced by permission of the Royal Society of Chemistry from [51]–Reproduced by permission of The Royal Society of Chemistry).

Silver nanoparticles (AgNPs) deposited on the surface of microporous polypropylene membranes (MPPMs) exhibit high catalytic activity in reduction of methylene blue (MB) in flow-through conditions [51] (Figure 10). 

In the flow-through membrane reactor, AgNPs-immobilized MPPM shows long-live catalytic activity, stability, recoverability and reusability. The MB conversion was equal to 60% after 2.5–4 h. Regeneration and activation of AgNPs-immobilized MPPM was performed by washing with ethanol. These examples clearly show that the membrane-based flow-through catalytic reactors are effective tool for decontamination of organic dyes from the wastewater. 

**Figure 11:**
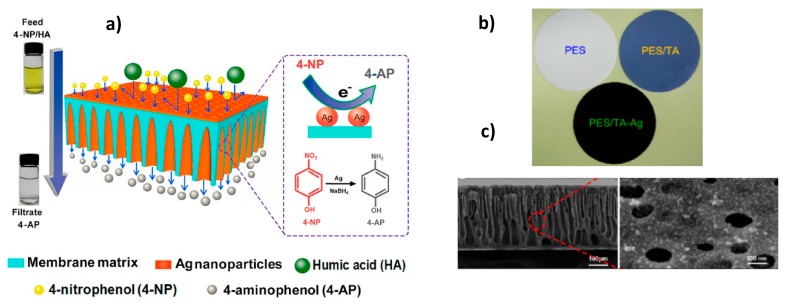
Scheme of membrane flow-through reactor for simultaneous catalysis and separation (**a**), PES, PES/TA and PES/TA-AgNPs membranes (**b**), cross-section of PES/TA-AgNPs membrane (**c**). (Reprinted from [52]).

Fabrication of polyethersulfone (PES) ultrafiltration membranes containing AgNPs and bearing simultaneously the function of catalyst and separation of the product is demonstrated in Figure 11 [52]. Polyphenol tannic acid (TA) in situ blended in the PES (PES/TA) played the role of both reducing agent of silver ions and stabilizer of AgNPs. 

Filtration performance of PES, PES/TA and PES/TA-AgNPs membranes with pore size in the range of 14.2–15.8 nm and porosity 93.2–94.7% were tested with respect to water flux, retention of humic acid (HA) and bovine serum albumin (BSA) while the catalytic activity of the same membranes was studied in the reduction of 4-NP. The pure water flux for pristine PES (255.6 L·m^−2^·h^−1^) increased for PES/TA up to 374.6 L·m^−2^·h^−1^ and decreased to 239.8 L·m^−2^·h^−1^ for PES/TA-AgNPs. The BSA and HA rejections were in the range of 84.3–96.1% and 62.3–87.3% respectively. Thus PES/TA-AgNPs membrane effectively removes both BSA and HA from aqueous solution. 

**Figure 12:**
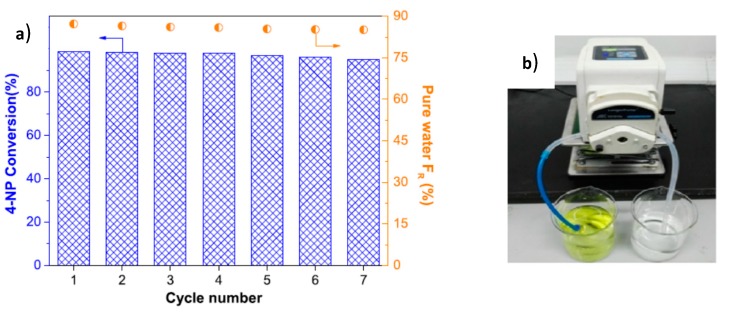
Conversion of 4-NP, pure water flux recovery ratio (F_R_) and filtration of HA with PES/TA-AgNPs membrane (**a**) after 7 times passing through membrane type catalytic reactor (**b**). (Compiled and reprinted from [52]).

The hydrogenation of 4-NP in the presence of PES, PES/TA and PES/TA-AgNPs membranes were compared in static (batch reactor) and dynamic (continuous reactor) regimes. PES and PES/TA membranes were inactive themselves while the PES/TA-AgNPs membrane exhibited high activity in the reduction of 4-NP. It should be stressed that the rate constant of PES/TA-AgNPs in the dynamic catalytic condition was 10^3^ times higher than the batch regime. Finally, the reduction of 4-NP and rejection of HA was performed in PES/TA-AgNPs membrane that acted simultaneously as flow-through catalytic reactor and separation filter. After 7 cyclic passing of aqueous solution of 4-NP and HA through the PES/TA-AgNPs membrane, the conversion of 4-NP reached 98%, pure water flux recovery ratio (F_R_) was at 85–87% and the rejection of HA was 89% implying the simultaneous reduction of 4-NP, water flux and removal of HA (Figure 12).

Elimination of N,N-diethyl-meta-toluamide (DEET) as a model pollutant was performed with iron oxide catalyst supported on powdered activated carbon and consequently deposited to ozone-resistant PVDF microfiltration and ultrafiltration membranes [53]. The hollow fiber reactor possessing large surface area/volume ratio (7000 m^−1^) and equipped with membrane distributor, contractor and separator is packed into a small volume making it cost-effective and attractive for industrial application. The PVDF membranes provided several functions: (1) adsorption and ozonization of pollutants, (2) concentrator of pollutants in the reactor, (3) producer of clean water and (4) gas distributor to generate fine ozone bubbles. The compact membrane reactor unit outperformed a semi-batch ozone reactor with 60% DEET conversion and 30% total organic carbon (TOC) reduction *versus* 20% DEET and 5% TOC. 

**Figure 13:**
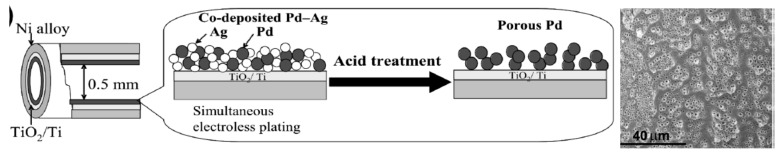
The scheme of Ag-Pd co-plating (**a**) and sequential removal of Ag to obtain porous Pd layer (**b**). (Reprinted from [15]).

Javaid et al. [15] developed tubular reactors with inner diameter less than 0.5 mm, the inner surface of which was uniformly coated by thin (1–2 µm) Pd, Pt and Rh layers by an electroless plating method (Figure 13). At first the bimetallic alloy Ag-Pd was deposited on the inner wall of tubular flow-through reactor. Then the Ag was leached out by continuous passing of 4M HNO_3_ leaving the porous Pd layer. 

Hydrogenation of 4-NP with formic acid in aqueous solution was carried out in tubular rector coated with Pd, porous Pd, bimetallic Ag-Pd and PdO at 30–40 °C, at fixed flow rate of 0.8 mL·min^−1^. The conversion of 4-NP increases in the following order: Ag-Pd>Pd> porous Pd>PdO> porous PdO. Rather high (>99%) conversion exhibits a porous PdO surface compared to corresponding nonporous precursors. The oxidized and porous PdO retain the catalytic activity without leaching of Pd and loss of efficiency after 100 h continuous operation.

Chiral calcium phenoxide (Ca(OR_2_)-pyridinebisoxazoline Pybox) complex was applied to asymmetric 1,4-addition reactions of 1,3-dicarbonyl compounds (1) with nitroalkenes (2) to obtain the γ-nitro carbonyl compounds (3) with high enantioselectivities (*ee*) in a continuous flow system [54]. As a result, a series of 1,4-addition products were obtained with high yields (ca. 92.4%), enantioselectivity (ca. 92.8% *ee*) and TON (228) during 8.5 days continuous flow without loss of activity.

Aerobic oxidation of benzyl alcohol in flow conditions was performed with the help of continuous ceramic membrane reactor inner part of which was impregnated with bimetallic Au-Pd catalyst [55]. The high catalytic activity (operation time is over 670 h), conversion degree (~25%) and selectivity to benzaldehyde (~97%) is probably due to improved oxygen mass transfer to the catalytic sites in contrast to a previously studied [56] packed-bed reactor with an Au-Pd/TiO_2_ catalyst. Further the Au-Pd/TiO_2_ system was used [57] for integration of kinetic models of benzyl alcohol oxidation merging (combining) the information obtained from batch glass stirred reactor (GSR) and continuous-flow micro-packed bed reactor (MPRB) experiments for an exact quantitative description of the products distribution. In fact, the kinetic models identified from GSR were used for the validation of MPRB. The results revealed a difficulty of estimation of kinetic parameters related to the disproportionation reaction of two molecules of benzyl alcohol limiting the selectivity to benzaldehyde.

**Figure 14:**
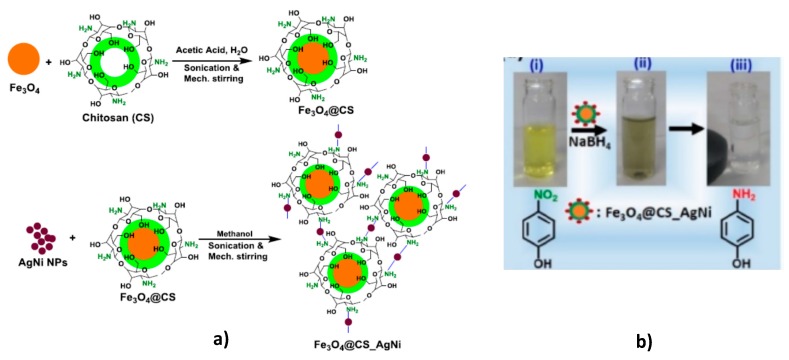
Synthetic protocol of preparation of Fe_3_O_4_@CS and Fe_3_O_4_@CS/AgNi core-shell structure (**a**) and reduction of 4-NP accompanied by separation of Fe_3_O_4_@CS/AgNi using an external magnet (**b**). (Reprinted from [58]).

Supporting of AgNi bimetallic nanoparticles on the surface of core-shell structure consisting of Fe_3_O_4_ and chitosan (Fe_3_O_4_@CS) leads to formulation of recoverable and reusable catalytic system for rapid reduction of nitroaromatic compounds to corresponding nitroamines [58] (Figure 14). 

Due to synergistic effect, bimetallic AgNi catalyst is better than monometallic Ag or Ni. This is attributed to the changes of geometric, electronic, and morphologic behavior of AgNPs caused by NiNPs. The values of apparent rate constants *k_app_* for Fe_3_O_4_@CS, Fe_3_O_4_@CS/Ni, Fe_3_O_4_@CS/Ag and Fe_3_O_4_@CS/AgNi are equal to 0.02 min^−1^, 0.03 min^−1^, 0.42 min^−1^ and 0.56 min^−1^ respectively. For the most substrates the conversion degree was equal to 100% excepting for nitroaniline (65%), o-chloronitrobenzene (70%), nitrobenzene (77%) and 2,4-dinitrophenol (90%). Thus, AgNi bimetallic nanoparticles deposited on core-shell structure of Fe_3_O_4_@CS representing a batch type catalyst exhibiting high efficiency, reusability and recoverability in the reduction of nitroarene compounds.

Efficiency of membrane supported metal nanoparticles in reduction of different substrates is summarized in Table 5. 

**Figure 15:**
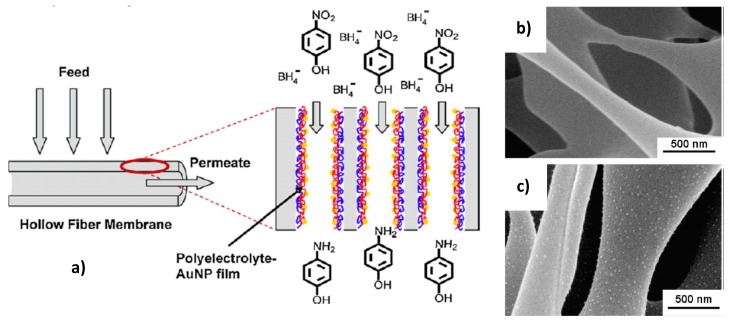
Modification of hollow fiber membrane using LbL technique (**a**) and SEM images of pristine PS (**b**) and coated (**c**) with a poly(styrene sulfonate)/poly(allylamine hydrochloride)/gold nanoparticles (PSS/PAH/AuNPs) samples. (Reprinted from [60]).

Layer-by-layer (LbL) deposition of metal nanoparticles within porous membranes provides a simple protocol for preparation, easy control over the deposited number of nanoparticles, a rapid mass transport of reactants to catalytic centers, high conversion degree, and easy separation of products from the feed etc. [13]. The surface of alumina membrane [59], hollow polysulfon (PS) and PES-based fiber microfiltration membranes [60] were modified by adsorption of polyelectrolyte-metal nanoparticles via LbL technique and afterwards used in the reduction of 4-NP with sodium boronhydride (Figure 15).

**Figure 16:**
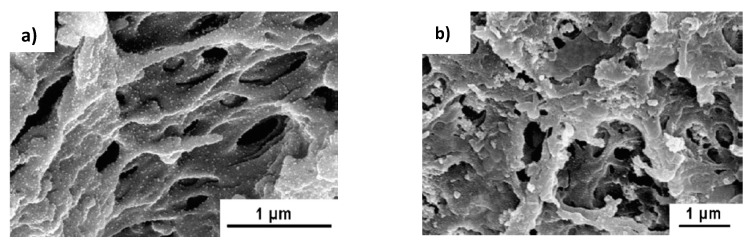
Morphology of PES-PSS/PAH/AuNPs before (**a**) and after (**b**) hydrogenation of 4-NP and percent reduction of 4-NP (**b**) after 3 h. (Reprinted from [60]).

The catalytic activity of PES- and PS-coated PSS/PAH/AuNPs results in 99% reduction of 4-NP while control experiments carried out with PSS/PAH yields less than 1% conversion under similar condition. Comparison of the efficiency of hollow fiber membranes modified by PSS/PAH/AuNPs with respect to 4-NP reduction shows that PS-coated PSS/PAH/AuNPs exhibits better results due to higher permeability. The morphology and catalytic activity of PES-PSS/PAH/AuNPs before and after hydrogenation of 4-NP is compared in Figure 16. It is seen that after cyclic exploitation of hollow membrane during 3 h the conversion of 4-NP decreases up to 60%, the initial structure of membrane changes (probably due to fouling), the fine distributed gold nanoparticles probably aggregate and by-products are deposited on the surface of membrane. Such phenomenon was also observed in the case of cryogel catalyst [41]. As distinct from PES-PSS/PAH/AuNPs the PS-PSS/PAH/AuNPs holds the activity at the level of 95–98% with minimal leaching of Au (<5 ppb) during the 4h of reaction. 

**Figure 17:**
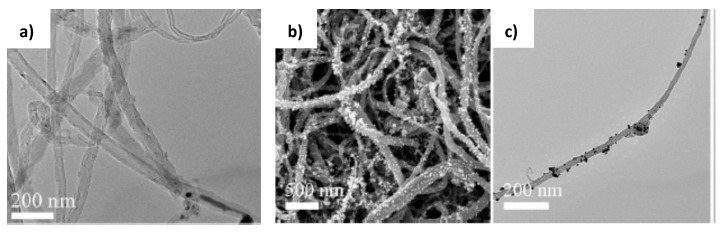
TEM of pristine CNT (**a**), SEM of HCNM-supported AuNPs (**b**), TEM of AuNPs-decorated CNT (**c**). (Reprinted from [61]).

The hierarchical carbon nanotube membrane (HCNM) decorated with AuNPs was supported on stainless steel mesh and used as flow-through catalytic reactor for hydrogenation of 4-NP [61] (Figure 17). The AuNPs were attached to HCNM using layer-by-layer (LbL) method. 

In batch experiments, HCNM-supported AuNPs retained 78% of catalytic activity compared to suspended AuNPs. In continuous flow-through conditions, HCNM-supported AuNPs showed 71% of the maximum catalytic activity under the batch configuration. Table 6 compares the first-order rate constants of 4-NP reduction obtained for HCNM-supported AuNPs and alumina membrane supported AuNPs. 

In the flow-through reactor the rate constant of alumina membrane-supported AuNPs is 3 times higher than HCNM-supported AuNPs in spite of similar size of AuNPs. This is probably attributed to small pores of alumina membrane that require a shorter time for diffusion of reagents to the catalytic sites but the small pores require a higher pressure to push the reaction mixture through small pores. In case of batch system, the influence of the pore size on the rate constant is negligible. Thus, LbL deposition of AuNPs is effective tool to fabricate hollow fiber catalytic membrane reactors.

**Figure 18:**
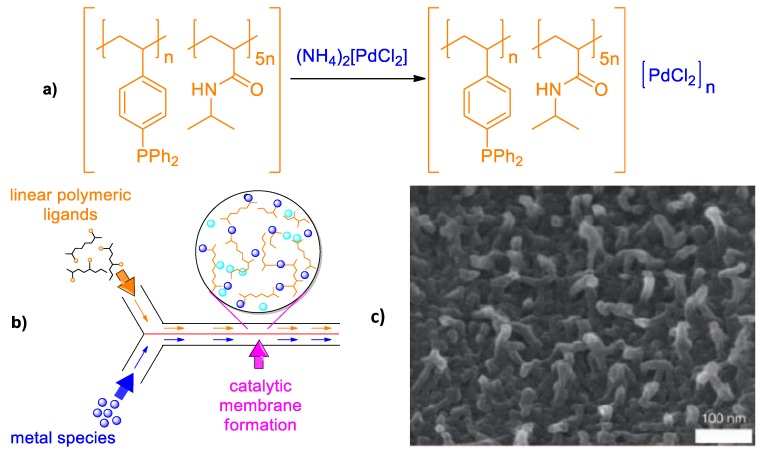
Polymer-metal catalyst composed of poly[(N-isopropylacrylamide)_5_-*co*-(4-diphenylstyrylphosphine)] and [PdCl_4_(NH_4_)_2_ (**a**), concept for preparation of catalytic membranes at the interface of a laminar flow inside a microchannel reactor (**b**) and high-resolution SEM image of membrane surface (**c**). (Reprinted from [62]).

A novel concept for catalyst immobilization into a glass microchannel catalytic reactor, or so called “convolution-convergent” approach, was introduced by Yamada et al. [62]. The sense of this invention is that a soluble polymer containing multiple ligand groups is convoluted with solution of transition metal ions and forms polymer-metal complexes stabilized by coordination or ionic bonds thus combining both heterogeneity and catalytic activity inside of a microchannel reactor with a Y-junction (Figure 18).

**Figure 19:**
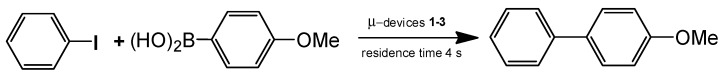
Suzuku-Miyaura reaction carried out by the catalytic membrane-installed microchannel devices 1-3. T = 50 °C, residence time 4 s, TOF 520 h^−1^. (Reprinted from [62]).

In this way the polymer-metal catalyst composed of poly[(N-isopropylacrylamide)_5_-*co*-(4-diphenylstyrylphosphine)] and [PdCl_4_(NH_4_)_2_ was prepared in ethyl acetate at 25 °C with a flow rate of 25 μL·min^−1^ and used as polymer membrane PA-TAP-Pd. The thickness of polymeric membrane adhered to the glassware of the microchannel was 1.3 μm, height is 40 μm, and length – 140 mm. For fabrication of palladium membrane-installed microchannel devices different polymeric ligands such as poly(4-vinylpyridine) (P4VP), poly(viologen) (PV) and PdCl_4_^−2^ were used. Three types of microchannel devices made of PA-TAP-Pd, P4VP-Pd and PV-Pd (μ-devices 1-3) were tested as catalysts for cross-coupling of aryl halides with aryl-boronic acids as exemplified in Figure 19. Totally 35 Suzuku-Miyaura reactions were studied and the corresponding coupling products quantitatively obtained within 5 and 1 s of residence time. In the future the function of such catalytic membrane reactor may diversely be expanded to other catalytic transformations. 

## 5. Conclusions

The flow-through catalytic reactors fabricated from porous gels and membranes are a new rapidly developing research area that can revolutionize many catalytic reactions, in particular, in pharmaceutical industry, where a high conversion and pure final product is required. The minimal volume of catalyst, high surface to volume ratio, energy saving and “green chemistry” aspects together with high productivity and low-cost principles are challenging target of flow-through catalytic reactions and reactors. The porous flow-through catalytic reactors can provide cascade type successive synthesis of target products by designing a flow set-up consisting of several flow reactors. The major drawback of cryogel microreactors is weak mechanical properties that can be overcome by forming interpenetrating networks or embedding clay minerals into the gel network resulting in improvement of their physico-mechanical characteristics. A serious problem in flow-through catalytic reactors represents the leaching out of metal nanoparticles from the 3D-network and membrane surfaces that can cause contamination of final products with metal nanoparticles. In some cases, such drawback can be overcome by application of hassle-free magnetic catalysts like Fe_3_O_4_-coated with polymer-protected metal nanoparticles as demonstrated in Ref. 58. Moreover, correlation between porosity and flow parameters, interconnectivity of pores, surface area, pore volume that are key characteristics for the design of highly efficient flow-through catalytic reactor is still lacking. Special interest may represent immobilization (or imprinting) of enzymes within the three-dimensional polymers, as monoliths, microcapsules and membranes [63]. Such approach can offer new monolith flow-through reactors that are several orders of magnitude catalytically more efficient and can be used for a long time in continuous process. A gentle combination of enzymes, mono- and bimetallic nanoparticles within nano-, micro- and macrosized polymeric gels and membranes can cause synergetic effect in flow-through catalytic reactions. 

## Figures and Tables

**Table 1:** Comparison of TONs and TOFs between PdNPs/MPGs, PdNPs/porous glass membrane, PdNPs/silica particles and PdNPs/C and Pd leaching.

Nanocatalyst	PdNPs/MPG-1	PdNPs/MPG-2	PdNPs/MPG-3	PdNPs/Porous Glass Membrane	PdNPs/Silica Particles	PdNPs/C
TON	2631	2290	1333	65	144	26
TOF, h^−1^ (t = 0.1 h)	27.4	16.1	7.8	-	-	-
Pd leaching, %	0	0	0	5.0	2.9	1.6

**Table 2:** Kinetic parameters of hydrogenation of nitroaromatic compounds and oxidation of DTT over macroporous flow-through catalytic reactors.

Macroporous Flow-Through Catalyst	Substrate	E_a_, kJ·mol^−1^	TON	TOF, h^−1^	Run	Ref.
DMAEM-MAA/AuNPs	4-NP	7.52	38.17	21.56	50	[38,40]
	DTT	-	985.2	412.2	10	[40]
	*p*-NBA	13.8	-	-	5	[41]
DMAEM-MAA/PdNPs	*p*-NBA	38.83	-	-	10	[41]
P4VP/CoNPs	*p*-NBA	18.9 ± 1.3		131.4	6	[31]
PVI/CoNPs		25.4 ± 1.8		82.2	8	

**Table 3:** Kinetic parameters of NaBH_4_ hydrolysis and hydrogenation of aromatic nitrocompounds over macroporous batch-type catalytic reactors.

Macroporous Batch-Type Catalyst	Substrate	E_a_, kJ·mol^−1^	TON	Run	Ref.
P(APTMACl)/[CuCl_4_]^−2^	NaBH_4_	61.9	-	-	[43]
P(APTMACl)/[CoCl_4_]^−2^		52.2	-	10	
P(APTMACl)/[NiCl_4_]^−2^		30.9	-	-	
P(SBMA) microgel/NiNPs	4-NP	35.64	-	3	[44]
P4VP/NiNPs	NaBH_4_	-	0.7 ± 0.2	-	[37]
P4VP/CoNPs		-	2.1 ± 0.4	-	

**Table 4:** Kinetic parameters of free and immobilized amyloglucosidase.

State of Enzyme	K*m*, mg·mL^−1^	*Vmax*, µmol·min^−1^
Free	2.743 ± 0.075	2.020 ± 0.059
Immobilized	0.865 ± 0.067	0.496 ± 0.054

**Table 5:** Catalytic activity of metal nanoparticles immobilized into different membrane surface with respect to various substrates.

Membrane Catalyst	Substrate	Conversion Degree, %	Flow Rate, mL·min^−1^*	Refs
PDMAEM-*b*-PS/ AuNPs/PVDF	4-NP	88–100	0.5	[49]
	Rhodamine B	91	0.5	
	Methyl orange	88	0.5	
MPPM/AgNPs	Methylene blue	60		[50]
PES/TA-AgNPs	4-NP	98	239.8 L·m^−2^·h^−1^	[51]
Ceramic/ Au-Pd	Benzaldehyde	25	-	[54]
Fe_3_O_4_@CS/AgNi	Nitroaromatic compounds	100	0.56	[57]
*Excepting the flow rate of PES/TA-AgNPs				

**Table 6:** Comparison of the first-order rate constants of 4-NP reduction catalyzed by suspended AuNPs and HCNM-supported AuNPs.

Catalyst	AuNPs Size, nm	Rate Constant *, µm·s^−1^	Pore Size, nm	Refs
AuNPs suspended in batch system				
Alumina membrane	12	140	~2·10^2^	[58]
HCNM	13.3 ± 2.4	111 ± 2	~1·10^4^	[60]
AuNPs supported in flow-through reactors				
Alumina membrane	12	180	~2·10^2^	[58]
HCNM	13.3 ± 2.4	62 ± 4	~1·10^4^	[60]
* The rate constants are normalized to the surface area of nanoparticles				
